# Violence against female sex workers in the state of Piauí, Brazil – a cross-sectional study

**DOI:** 10.1038/s41598-025-22139-3

**Published:** 2025-11-03

**Authors:** Afonso Ribeiro Alves-filho, Nikolaos Angelakopoulos, Anna Turkina, Ademir Franco

**Affiliations:** 1 Division of Anatomy and Forensic Odontology, Faculdade São Leopoldo Mandic, Campinas, Brazil; 2 Department of Orthodontics and Dentofacial Orthopedics, University of Bern, Freiburgstrasse 7, Bern, 3010 Switzerland; 3 Department of Therapeutic Stomatology, Sechenov University, Moscow, Russia

**Keywords:** Forensic dentistry, Crime, Sexual violence, Physical violence, Psychological violence, Women’s health, Female sex workers, Health care, Medical research, Psychology, Psychology, Risk factors

## Abstract

By quantitatively assessing the exposure of female sex workers to violence in Piauí, Brazil, this study aimed to identify associated risk factors and contribute evidence-based public health strategies and human rights policies, advancing health and gender equality. This was a quantitative, observational, analytical, cross-sectional study. A structured questionnaire was administered to 218 female sex workers, aged 19 to 57 years (mean age = 31.76 ± 8.33 years), who were actively working in the state of Piauí, Brazil. The questionnaire addressed demographic characteristics, work-related practices, and exposure to psychological, physical, and sexual violence. Odds ratios (ORs) for the association between variables and the exposure to violence were calculated. Most participants had incomplete higher education (54.2%), self-identified as Black (42.7%), were single (77.4%), heterosexual (89.8%), and mothers (71.2%). Approximately 50% of participants reported being exposed to some form of violence, with physical violence being the most prevalent (75.7%), followed by psychological (43.0%) and sexual violence (12.2%). The odds of exposure to violence were inversely associated with educational attainment (*p* = 0.026) and were significantly related to marital status (*p* = 0.031), having children (*p* < 0.001), and being pregnant (*p* = 0.001). These findings demonstrate the vulnerability of female sex workers to violence in the State of Piauí, Brazil, and the need for tailored public policies that could also benefit society as a whole, such as promotion to scholar education and sexual health awareness.

## Introduction

The Sustainable Development Goals (SDGs), adopted by United Nations member states, have been in effect for a decade. These seventeen globally interconnected objectives encompass 169 specific targets^1^. Violence against women, particularly against sex workers, is directly linked to several of these goals, including SDG 3 (Good Health and Well-being), SDG 4 (Quality Education), SDG 5 (Gender Equality), SDG 8 (Decent Work and Economic Growth), SDG 10 (Reduced Inequalities), and SDG 16 (Peace, Justice, and Strong Institutions). Although violence against women is widely acknowledged as a pressing public health and security issue^2^, it remains substantially underreported, especially within marginalized populations. The inherently precarious nature of sex work increases the vulnerability of sex workers to violence. Moreover, they are frequently marginalized within society^3^ and consequently neglected by governmental protection policies and safety interventions^2,4^.

Physical, psychological, and sexual violence are the most commonly reported forms in the scientific literature^3,5–9^ and are formally recognized by the World Health Organization^10^. Alternative forms of violence may be classified according to their context, such as domestic and occupational violence. Female sex workers are vulnerable to both: domestic violence typically originates from intimate partners within the home, whereas occupational violence is usually perpetrated by clients^3^. From an epidemiological perspective, violence against sex workers occurs at alarmingly high rates globally^4,11^, with particularly heightened incidences in specific contexts, such as during the COVID-19 pandemic^12^ and among migrant workers displaced by civil crises^13^.

Internationally, institutions such as the Global Network of Sex Work Projects (NSWP) advocate for the rights of sex-workers (https://www.nswp.org/). As the lead part in a consortium of Sex Worker Networks, NSWP has promoted sex work understanding, organization and regulation covering five geographic regions, including affiliated members in Latin America. Among their multiple interdisciplinary contributions is the implementation and dissemination of the term “sex workers” to endorse the professional aspect of the practice. In Brazil, sex work has been officially recognized as a profession^3^. The pursuit of rights by female sex workers dates back to at least 1987, following a national meeting that catalyzed organized movements, including the Group of Women Prostitutes of the State of Pará (GEMPAC)^14^. National examples of organized advocacy include the Brazilian Network of Prostitutes (RBP), the National Association of Sex Workers (ANPROSEX), and the Unified Female and Male Sex Workers’ Central (CUTS). At the regional level, sex workers are supported by the Latin American and Caribbean Network of Female Sex Workers (RedTraSex). These institutions have advocated for improved working conditions and enhanced protection for sex workers. Notably, the Association of Prostitutes of the State of Piauí (APROSPI) has been officially recognized as a municipal public utility institution under Law n. 5.626/2021.

Several studies sampling female sex workers have been conducted in Brazil. For example, performed a qualitative study involving eleven women affiliated with APROSPI, revealing an occupational environment characterized by humiliation, theft, threats, breaches of agreements, and false accusations^15^. Similarly, Arruda-Barbosa et al. (2024) conducted a qualitative investigation of Venezuelan sex workers in Brazil, documenting severe violence and exploitation^13^. While quantitative studies have also been conducted in the state, data collection for these was completed over a decade ago^16,17^. A prior study involving over 2,500 female sex workers across ten cities has been conducted^18^; however, the state of Piauí was not included. Despite the significant contributions of existing research, further studies remain necessary to comprehensively understand and monitor the situation in this region.

As a contribution to the existing body of knowledge, this study aimed to quantitatively assess the exposure of female sex workers to violence in the State of Piauí, Brazil, and to identify associated risk factors to strengthen evidence-based public health strategies and human rights policies, advancing health and gender equality.

## Methods

### Study design and ethical aspects

An observational, analytical, cross-sectional study was conducted. Ethical considerations adhered to the principles outlined in the Declaration of Helsinki (revised 2024), and approval was obtained from the Institutional Committee of Ethics in Human Research (protocol number 79616524.9.0000.5374). The study was partially reported in accordance with the Enhancing the Quality and Transparency of Health Research (EQUATOR) network guidelines, specifically following the Strengthening the Reporting of Observational Studies in Epidemiology (STROBE) statement^19^.

### Sample and participants

Sample selection was based on predefined eligibility criteria. Inclusion criteria required participants to be female, Brazilian sex workers over 18 years of age, actively engaged in sex work within the state of Piauí (Northeastern Brazil). Exclusion criteria included illiteracy, failure to provide informed consent, incomplete questionnaire responses, and lack of affiliation with APROSPI. The sample size was determined by referencing the questionnaire-based study by Aristegui et al. (2022),which featured similar quantitative methods and reported an overall non-response rate of 27.9%^20^. In this context, the target population was estimated at 600 women affiliated with APROSPI. A sample size calculation was performed using an 80% confidence level and a 5% margin of error. The initial estimated sample size was 130 participants, which was increased to 166 to account for the anticipated non-response rate. An additional buffer of 10 participants was included to accommodate potential dropouts or incomplete responses during data collection. Therefore, the final minimum estimated sample size was set at 176 participants.

### Settings and variables

A survey was conducted using a self-administered questionnaire presented in hard copy and completed in person. The questionnaire included items that have been adapted from previous studies on violence against women and/or sex workers^7,18,20^. Participants were approached at APROSPI’s office and related facilities and completed the questionnaire individually in a private room, supervised by a trusted female member of APROSPI. The questionnaire was initially developed by a public health expert and subsequently refined by APROSPI’s lead representative. It was designed to collect data on the following variables: (1) level of education, (2) self-declared ethnicity, (3) marital status, (4) sexual orientation, (5) parenthood status, (6) pregnancy status, (7) exposure to violence, (8) history of examination at the medicolegal institute following violent incidents, and (9) condom use with clients.

Data collection was conducted over a six-month period, between July/2024 and January/2025, in the cities of Teresina and Picos, both located in the state of Piauí, Brazil. Approximately 600 sex workers are registered in the association and data collection was accomplished through sequential approach of female sex workers available for interview. The collected data were entered into an Excel spreadsheet (Microsoft Corp., Redmond, WA, USA) for subsequent statistical analysis.

### Statistical analysis

Categorical variables collected through the questionnaire were analyzed using descriptive statistics, reporting absolute (n) and relative (%) frequencies. Subsequently, associations between each variable and the odds of exposure to violence were evaluated using logistic regression models to estimate odds ratios (OR) with corresponding 95% confidence intervals (CIs). The OR was calculated based on associations between variables and the outcomes (exposure to violence), and did not reflect causation. P-values were derived from Wald tests. All statistical analyses were performed using Stata software, version 15 (StataCorp LLC, College Station, TX, USA), with a significance threshold set at 0.05.

## Results

The sample comprised 218 female sex workers who completed the questionnaire. The majority were from the capital city of Teresina (*n* = 208), with the remaining 10 participants from Picos. Participants’ ages ranged from 19 to 57 years, with a mean of 31.76 ± 8.33 years. Most women had incomplete higher education (54.2%), self-identified as Black (42.7%), were single (77.4%), heterosexual (89.8%), and mothers (71.2%). While the majority reported not being pregnant, 21.3% indicated they were pregnant at the time of data collection. Regarding healthcare experiences, nearly all participants reported never having undergone a forensic medical examination at a medicolegal institute following an episode of violence. Condom use with clients was reported by 94% of the sample (Table 1).

**Table 1:** General characteristics of the female sex workers addressed in this study.

Level of education	*n* (%)
Incomplete primary education	56 (25.9)
Incomplete secondary education	43 (19.9)
Incomplete higher education	117 (54.2)
Self-declared ethnicity	
White	62 (29.1)
Mixed	60 (28.2)
Black	91 (42.7)
Marital status	
Married/stable union	10 (4.6)
Divorced	20 (9.2)
Single	168 (77.4)
Widow	19 (8.8)
Sexual orientation	
Heterosexual	193 (89.8)
Homosexual	4 (1.9)
Bisexual	16 (7.4)
Other	2 (0.9)
Children	
No	62 (28.8)
Yes	153 (71.2)
Pregnant	
No	166 (78.7)
Yes	45 (21.3)
Exposure to work-related violence	
No	108 (50.2)
Yes	107 (49.8)
Examined in a medicolegal institute	
No	102 (95.3)
Yes	5 (4.7)
Use condoms	
No	13 (6.1)
Yes	202 (93.9)

*n* Absolute frequency, *%* Relative frequency. Incomplete higher education being either interruption (drop-out) or enrollment without completion (ongoing studies).

Among the 107 women who reported being exposed to some form of violence, 75.7% indicated physical aggression, 43% psychological aggression, and 12.2% sexual aggression. Furthermore, 24.2% of those affected reported exposure to at least two types of violence, while 6.5% have been exposed to all three forms. Among the women who reported any prior exposure to violence, only 27 (12.4%) disclosed the incident. When asked about the perpetrators’ sex, 45% identified females, 39.6% males, and 15.4% reported both female and male perpetrators. Regarding the number of perpetrators, the majority (71.8%) indicated a single perpetrator, while 28.2% were exposed to violence from two or more individuals.

The odds of exposure to violence were 62% and 56% lower among women with incomplete secondary education and incomplete higher education (meaning either interruption/drop-out or enrollment without completion/ongoing), respectively, compared to those with incomplete primary education (*p* = 0.026). Exposure to violence was also significantly associated with marital status; widowed women had nearly tenfold higher odds of being exposed to violence (OR = 9.80; 95% CI = 1.50–63.85) compared to married women or those in stable unions. Furthermore, the odds of exposure to violence were 3.3 times higher among women who had children (*p* < 0.001) and among those who reported being pregnant (*p* = 0.001) (Table 2).

**Table 2:** Association between exposure to violence and the studied variables, followed by their odds.

	Exposure to violence *n* (%)	OR (CI_95%_)	*p*
Level of education			0.026
Incomplete primary education	36 (65.5)	1.00	
Incomplete secondary education	18 (41.9)	0.38 (0.17–0.87)	
Incomplete higher education	53 (45.7)	0.44 (0.23–0.86)	
Self-declared ethnicity			0.953
White	30 (48.4)	1.00	
Mixed	29 (49.2)	1.03 (0.51–2.10)	
Black	46 (51.1)	1.12 (0.58–2.13)	
Marital status			0.031
Married/stable union	2 (22.2)	1.00	
Divorced	7 (35.0)	1.89 (0.31–11.64)	
Single	84 (50.3)	3.54 (0.72–17.55)	
Widow	14 (73.7)	9.80 (1.50–63.85)	
Sexual orientation			1.000
Heterosexual	8 (50.0)	1.00	
Homosexual	96 (50.0)	0.52 (0.36–2.77)	
Bisexual	2 (50.0)	1.12 (0.11–8.95)	
Other	0 (0.0)	-	
Children			< 0.001
No	18 (29.5)	1.00	
Yes	88 (57.9)	3.29 (1.74–6.21)	
Pregnant			0.001
No	74 (44.9)	1.00	
Yes	32 (72.7)	3.28 (1.58–6.81)	
Examined in a medicolegal institute			-*
No	101 (100.0)	-*	
Yes	5 (100.0)	-*	
Use condoms			0.408
No	8 (61.5)	1.00	
Yes	98 (49.0)	0.60 (0.19–1.90)	

*The question was only displayed to participants who answered “yes” to exposure to violence.

## Discussion

The present study examined violence against female sex workers in Brazil, specifically in the Northeastern state of Piauí. Approximately 50% of the surveyed women reported being exposed to violence in their occupational context. This prevalence aligns closely with findings by Kloek et al. in the Netherlands, who reported a 41% prevalence of violence^12^. Although there are methodological differences because Kloek et al.^12^ focused on violence during the COVID-19 pandemic and included a broader gender spectrum, the similarities underscore the widespread nature of violence affecting sex workers internationally. In contrast, their reported rates of physical violence (27%) and sexual violence (28%) were roughly equal, whereas the present study observed a higher prevalence of physical violence. Specifically, physical violence alone, manifested through various forms of aggression, was reported by 51.4% of the participants. The combination of physical and psychological violence accounted for 14% of cases, while the combination of physical, psychological, and sexual violence represented 6.5%. One possible explanation for this discrepancy lies in the differing structural conditions faced by female sex workers in Brazil. Many operate in informal settings with limited legal protections and face significant social stigma, factors that likely increase their vulnerability to physical and psychological violence. By contrast, the Netherlands has established more robust regulations recognizing sex work as a legitimate occupation (https://business.gov.nl/self-employed-sex-worker*)*, including labor rights that serve as protective measures against severe or repeated violence. With enhanced autonomy and legal safeguards, sex workers in the Netherlands may have greater capacity to operate independently and engage in entrepreneurial activities.

Other studies have addressed the topic of violence against sex workers covering a broad variety populations, such as from Argentina^20^, Nepal^2^ and India^6^. A systematic review published in 2014 has indicated that the prevalence rates of any or combined types of workplace violence against sex workers (not only females) were between 45% and 75%^4^. The present study’s outcomes fall within this range highlighting the persistence of violence as a global hazard for sex workers, regardless of geographical context. Differences, however, may be observed across countries and could be an expression of cultural, religious and (legal) regulatory characteristics in the target population studied.

Scientific literature has consistently shown that perpetrators of violence against female sex workers are predominantly male clients in cases of sexual aggression^21^, although intimate partners^18^, police officers, and other men also contribute to the violence^21^. A notable finding in the present study, however, was the high proportion of violence attributed to female perpetrators, accounting for 45% of reported cases. This finding suggests that female sex workers may be vulnerable not only to violence from clients but also from their peers. Such dynamics have been documented previously, with studies attributing this behavior to efforts to maintain territory and assert social status among sex workers^22^. Competition over clients can particularly elevate the incidence of psychological violence, such as threats, as well as physical aggression. These patterns are often more pronounced in countries with high levels of violence or in contexts where sex work remains inadequately regulated. In Brazil, brothels currently lack a specific legal framework to regulate their operation. On one hand, these establishments are perceived by some as potentially providing a more controlled environment in terms of safety and financial stability. On the other hand, they are also regarded as possible sites of exploitation of women^23^. Within this complex context, occupational organizations such as APROSPI play a critical role in protecting female sex workers and fostering safer working conditions. The findings of this study may have been significantly more severe in the absence of such protective initiatives.

The statistical analysis of potential risk factors associated with violence against female sex workers revealed significant associations with education level, marital status, having children, and being pregnant. These findings align with prior research conducted in Brazil, which identified lower educational attainment as a key predictor of violence, particularly in cases involving intimate partner aggression^18^. Lower levels of education may limit access to information, reduce awareness of rights, and constrain women’s ability to seek help or avoid high-risk situations, thereby increasing their overall vulnerability. Reduced education can be detrimental to working conditions, capacity to negotiate with clients, and understanding of legal rights. It may also hinder financial literacy, restricting the ability to plan, save, or effectively engage with formal economic systems. This, in turn, can increase dependence on exploitative relationships and reduce negotiating power, both in professional and domestic settings. As a result, women with lower levels of education may find themselves in more precarious and abusive environments, further amplifying their vulnerability to violence. Interestingly, more than 50% of the respondents demonstrated some level of formal education by reporting “incomplete higher education”. This contrasts with the overall rates for the State of Piauí. Data from the 2022’s demographic census released by the Brazilian Institute of Geography and Statistics indicated that within the federative units, Piauí exhibited the highest proportion of individuals aged 25 years and older with no formal or incomplete primary education (https://agenciadenoticias.ibge.gov.br/) – a situation that represents 49% of individuals in the State. However, because of the cross-sectional model of the present study, it is not possible to infer if women are quitting higher educational levels because of the nature of the work and its associated risks to violence, or if they managed to have access and afford (ongoing) higher education because of their work.

With regard to marital status, higher odds of being exposed to violence were observed among women who were not married or in a stable union. This does not necessarily indicate the absence of intimate partners; rather, these women may have engaged in more informal or multiple partnerships, which can also carry significant risks. These findings suggest that female sex workers are vulnerable to intimate partner violence regardless of their marital status.

Furthermore, the data showed that a majority of participants had children (71.2%), and a notable proportion were pregnant at the time of data collection (21.3%). Irrespective of pregnancy intentionality, female sex workers are particularly vulnerable to unintended pregnancies, partly due to the underuse of contraceptive methods—especially those controlled by women^24^. While the high prevalence of condom use observed in this study may indicate a strong awareness of the need to prevent sexually transmitted infections, it does not necessarily reflect comprehensive reproductive planning. Given that condoms are often partner-dependent, they may offer limited protection against unplanned pregnancy. This underscores the importance of increasing access to and education about autonomous contraceptive methods, alongside the provision of reproductive health care services tailored to the unique circumstances of female sex workers.

Some limitations of the present study should be acknowledged and addressed in future research. A primary limitation concerns the geographical scope of the sample, which was restricted to female sex workers operating in a single State of Brazil (Piauí). As a result, the findings may not be fully generalizable to other regions of the country or to contexts with differing socio-economic, legal, or cultural conditions. Broader studies encompassing multiple states or national samples would enhance representativeness and provide a more comprehensive understanding of the patterns and predictors of violence affecting this population. Moreover, the study did not explore certain sex-specific variables that could influence vulnerability to violence, such as the use of female-controlled contraceptive methods, duration of marriage or stable unions, and length of time engaged in sex work. Additionally, the current study was designed based on self-reporting through a questionnaire. While this approach allows participants to share and tell their own history and life-experiences, it is also susceptible to recall and underreporting, especially when it comes to sensitive topics such as exposure to violence. Finally, a limitation that deserves attention is the cross-sectional study model, which can enable statistical associations but no causality between variables. To this end, a cohort study would be optimal to monitor and assess sex workers’ exposure to violence. Despite these limitations, the findings of the present study offer meaningful insights that can inform the development of educational and protective strategies aimed at supporting female sex workers in their daily lives. While such strategies should be prioritized within governmental public health and safety policies, they may also serve as a valuable resource for professional associations, such as APROSPI and similar organizations, in their ongoing efforts to safeguard the health, rights, and well-being of women in this occupation.

## Conclusion

The female sex workers surveyed in this study were predominantly single, heterosexual, self-identified as Black, mothers, and had incomplete higher education. Approximately half of the participants reported being exposed to at least one form of violence, with physical violence being the most prevalent, followed by psychological and sexual violence. Higher odds of exposure to violence were significantly associated with lower educational attainment, lack of a marital or stable partnership, having children, and being pregnant. Notably, a substantial proportion of the reported violence was perpetrated by women, suggesting a complex interpersonal dynamic within this population. Although the present study does not represent a benchmark given the regional implication of its findings, the presented outcomes may serve as informative resource to help develop protective strategies and promote safer environments for women in this vulnerable social group.

## Data Availability

The data supporting this study’s findings are available on request from the corresponding author.

## References

[1] United Nations. Transforming our world: the 2030 agenda for sustainable development [Internet]. New York: United Nations. (2015). Available from: https://sdgs.un.org/2030agenda

[2] Beaujolais, B. et al. Client-perpetrated violence toward female sex workers in Kathmandu. *Violence against Women*. **26** (2), 249–267. 10.1177/1077801219832117 (2020). 30843761 10.1177/1077801219832117

[3] Costa, M. O. et al. Narratives of sex workers: intimate partner violence and coping strategies. *Revista Brasileira De Enfermagem*. **77** (6), e20240180. 10.1590/0034-7167-2024-0180 (2024). 39699369 10.1590/0034-7167-2024-0180 PMC11654522

[4] Deering, K. N. et al. A systematic review of the correlates of violence against sex workers. *Am. J. Public Health*. **104** (5), e42–e54. 10.2105/AJPH.2014.301909 (2014). 24625169 10.2105/AJPH.2014.301909 PMC3987574

[5] Evens, E. et al. Experiences of gender-based violence among female sex workers, men who have sex with men, and transgender women in Latin America and the caribbean: A qualitative study to inform HIV programming. *BMC Int. Health Hum. Rights*. **19**, 9. 10.1186/s12914-019-0187-5 (2019). 30832664 10.1186/s12914-019-0187-5 PMC6399914

[6] Javalkar, P. et al. What determines violence among female sex workers in an intimate partner relationship? Findings from North Karnataka, South India. *BMC Public. Health*. **19**, 350. 10.1186/s12889-019-6673-9 (2019). 30922283 10.1186/s12889-019-6673-9 PMC6440026

[7] Magrin, J. V., Franco, A., Makeeva, I., Paranhos, L. R. & Rigo, L. Emotional, physical and sexual violence against female students undergoing medical, dental and psychology courses in South Brazil. *Eur. J. Dent. Educ.* **23** (4), 455–460. 10.1111/eje.12452 (2019). 31274215 10.1111/eje.12452

[8] Nascimento, C. T. et al. Domestic violence against women detected and managed in dental practice: A systematic review. *J. Family Violence*. **38** (1), 149–160. 10.1007/s10896-021-00351-9 (2023). 10.1007/s10896-021-00351-9 PMC8732966 35013643

[9] Nascimento, C. T. et al. Knowledge and attitudes of rural healthcare providers regarding domestic violence against women: A systematic review. *São Paulo Med. J.* **142** (3), e2022682. 10.1590/1516-3180.2022.0682.R1.180723 (2023). 38055422 10.1590/1516-3180.2022.0682.R1.180723 PMC10703492

[10] World Health Organization. *Violence against women*. (2016). Available from: http://www.who.int/mediacentre/factsheets/fs239/en/

[11] McBride, B. et al. Underreporting of violence to Police among women sex workers in canada: amplified inequities for im/migrant and in-call workers prior to and following end-demand legislation. *Health Hum. Rights*. **22** (2), 257–270 (2020). https://www.ncbi.nlm.nih.gov/pmc/articles/PMC7762889 33390711 PMC7762889

[12] Kloek, M., de Vlas, S. J. & Hontelez, J. A. C. High rates of violence and low Police reporting amongst sex workers in the Netherlands during the COVID–19 pandemic. *Crime. Sci.* **13**, 38. 10.1186/s40163-024-00237-3 (2024).

[13] Arruda-Barbosa, L., Menegatti, M. S., Fonseca, R. M. G. S. & Oliveira, M. A. C. Violence suffered by Venezuelan immigrant female sex workers: an intersectional view. *Revista Da Escola De Enfermagem Da USP*. **58**, e20230282. 10.1590/1980-220X-REEUSP-2023-0282en (2024). 10.1590/1980-220X-REEUSP-2023-0282en PMC11126236 38743955

[14] Beltrão, J. F. & Bispo, A. F. Sexual work in brazil: an approach to the protagonism of prostitutes in their struggle for recognition of the right to exercise the profession. *Saúde Em Debate*. **47** (Especial 1), e8507. 10.1590/2358-28982023E18507I (2023).

[15] Moreira, I. C. C. & Monteiro, C. F. S. The violence in everyday of prostitution of women: invisibility and ambiguities. *Rev. Latinoam. Enferm.* **20** (5), 1–7 (2012). https://www.eerp.usp.br/rlae 10.1590/s0104-11692012000500018 23174841

[16] Penha, J. C. et al. Characterization of physical violence experienced by prostitutes living inside the state of Piauí. *Revista Brasileira De Enfermagem*. **65** (6), 984–990. 10.1590/S0034-71672012000600015 (2012). 23559178 10.1590/s0034-71672012000600015

[17] Penha, J. C. et al. Risk factors for sexually transmitted diseases among sex workers in the interior of Piauí, Brazil. *Revista Gaúcha De Enfermagem*. **36** (2), 63–69. 10.1590/1983-1447.2015.02.52089 (2015). 26334410 10.1590/1983-1447.2015.02.52089

[18] Lima, F. S. S., Merchán-Hamann, E., Urdaneta, M., Damacena, G. N. & Szwarcwald, C. L. Factors associated with violence against female sex workers in ten Brazilian cities. *Cad. Saúde. Públ.* **33** (2), e00157815. 10.1590/0102-311X00157815 (2017). 10.1590/0102-311X00157815 28380126

[19] Von Elm, E. et al. The strengthening the reporting of observational studies in epidemiology (STROBE) statement: guidelines for reporting observational studies. *J. Clin. Epidemiol.* **61** (4), 344–349. 10.1016/j.jclinepi.2007.11.008 (2008). 18313558 10.1016/j.jclinepi.2007.11.008

[20] Aristegui, I. et al. Female sex workers and Police violence during the Covid-19 health crisis in 2020–2021: results from the EPIC multi-country community-based research program in Argentina. *Harm Reduct. J.* **19** (1), 139. 10.1186/s12954-022-00714-5 (2022). 36503497 10.1186/s12954-022-00714-5 PMC9742027

[21] Jewkes, R. et al. Sexual IPV and non-partner rape of female sex workers: findings of a cross-sectional community-centric National study in South Africa. *SSM - Mental Health*. **1**, 100012. 10.1016/j.ssmmh.2021.100012 (2021). 10.1016/j.ssmmh.2021.100012 PMC8654680 34957423

[22] Kelly-Hanku, A. et al. Perpetration of violence by female sex workers in Papua new guinea: ‘We will crush their bones’. *Br. J. Criminol.* **61** (1), 104–122. 10.1093/bjc/azaa058 (2020). 35923353 10.1093/bjc/azaa058 PMC9345598

[23] Incao, M. C. & Seabra, A. Regulamentação do Trabalho sexual no Brasil. *Revista Ciências Do Trabalho*. **26**, 1–19 (2024). [Portuguese].

[24] Zemlak, J. L. et al. Interpersonal violence and contraceptive method use by women sex workers. *Women’s Health Issues*. **31** (6), 516–522. 10.1016/j.whi.2021.08.001 (2021). 34493434 10.1016/j.whi.2021.08.001

